# Effect of Comprehensive Health Management on Medication Adherence and Healthy Lifestyle Behavior of Patients With Hypertension

**DOI:** 10.1155/ijhy/1165809

**Published:** 2025-07-19

**Authors:** Xinyuan Lu, Jiwei Wang, Sikun Chen, Lin Lv, Jinming Yu

**Affiliations:** Key Laboratory of Public Health Safety Ministry of Education, National Health Commission Key Laboratory of Health Technology Assessment, School of Public Health, Fudan University, Shanghai, China

**Keywords:** comprehensive health management, healthy lifestyle behavior, hypertension, medication adherence

## Abstract

Suboptimal medication adherence and unhealthy lifestyle behaviors are well-recognized contributing factors to poor blood pressure control in hypertension patients. We evaluated the effectiveness of comprehensive health management in improving medication adherence and promoting healthy lifestyle behaviors among hypertension patients in China. A cluster randomized trial was implemented in rural areas of three provinces of China. Participants were individuals aged ≥ 40 years with uncontrolled hypertension. The intervention group received multidimensional health management measures codeveloped by healthcare organizations, village doctors, and patients, while the control group received standard care. The coprimary outcomes included the proportion of patients demonstrating good medication adherence and adherence to ≥ 3 healthy lifestyle components. Secondary outcomes comprised the proportion achieving controlled hypertension (BP < 140/90 mm Hg). From May 8th to November 28th, 2018, 9204 participants were enrolled. At 18-month follow-up, significantly higher medication adherence was observed in the intervention group compared with the control group, with an absolute difference of 5.0% (95% confidence interval (CI): 2.8–7.2; *p* < 0.001). Similarly, adherence to ≥ 3 healthy lifestyles was achieved by 45.8% in the intervention group versus 33.7% in controls, yielding a 12.1% between-group difference (95% CI: 9.9–14.3; *p* < 0.001). Hypertension control rates differed significantly between groups (43.2% vs. 23.9%; absolute difference 19.2% and 95% CI: 17.2–21.3; *p* < 0.001). Hypertension patients receiving comprehensive health management in rural China demonstrated superior medication adherence and healthier lifestyle behaviors compared with those receiving standard care over 18 months. Further investigations are warranted to evaluate the cost-effectiveness and generalizability of this intervention.

**Trial Registration:** ClinicalTrials.gov identifier: NCT03527719

## 1. Introduction

Hypertension affects approximately 27.5% of adults in China and remains a predominant risk factor for stroke and ischemic heart disease—the first and second leading causes of mortality and disability-adjusted life years (DALYs) nationally over the past 3 decades [1, 2]. Since 2009, China has launched the National Basic Public Health Service Project, integrating hypertension management as a core component of chronic disease care. Concurrently, a comprehensive policy framework has been established to standardize primary-level hypertension prevention and management [3]. Nevertheless, population-level hypertension treatment and control rates remain suboptimal. Epidemiological evidence indicates that in 2018, only 34.9% of Chinese adults with hypertension received treatment, with merely 11.0% achieving blood pressure control—substantially lower than global averages (treatment: 47% women/38% men and control: 23% women/18% men) [2, 4]. Effective hypertension management requires multifaceted coordinated strategies, including lifestyle modification, regular healthcare engagement, and guideline-directed antihypertensive therapy when indicated [5].

Medication adherence serves as a pivotal determinant in hypertension management. Substantial evidence demonstrates that improved adherence is positively correlated with blood pressure control, reduced global disease burden, and diminished risks of cardiovascular complications including stroke, myocardial infarction, and heart failure [6–9]. However, current hypertension medication adherence rates remain suboptimal worldwide. A landmark meta-analysis encompassing 27 million patients revealed a global prevalence of antihypertensive nonadherence ranging from 27% to 40% [10]. In China, the hypertension medication adherence rate was reported at merely 42.5% in mainland populations [11]. Maintaining a healthy lifestyle, or engaging in heart-healthy behavioral changes, also has a documented effect on lowering or controlling blood pressure and is recommended in guidelines for hypertension management in multiple countries [12–15]. Several healthy lifestyle behaviors have been confirmed as having significant effect on lowering blood pressure, including reducing sodium intake, maintaining a normal weight, avoiding or limiting alcohol intake, and engaging in regular physical activity [16–19]. While smoking cessation may not directly lower blood pressure, its cardiovascular risk reduction benefits warrant inclusion in hypertension lifestyle recommendations [12]. Alarmingly, adherence to healthy lifestyles remains inadequate among hypertensive populations. A nationwide Iranian study found that only 27.9% of patients maintained optimal lifestyle practices [20]. Similarly, recent data from Shenzhen, China, revealed low compliance rates: 38.7% for physical activity, 10.6% for dietary adherence, and 41.1% for normal BMI maintenance among hypertensive individuals [21]. These findings underscore the urgent need to develop innovative strategies for enhancing both pharmacological adherence and behavioral modification in hypertension care—a critical public health priority requiring multidisciplinary solutions.

Hypertension control faces multilevel barriers spanning patient, provider, healthcare system, and community domains, necessitating coordinated interventions across these levels to enhance medication and lifestyle adherence [22–24]. The American Heart Association's scientific statement on medication adherence emphasizes that effective management demands collaborative engagement from individual patients through healthcare systems [25]. A cluster randomized controlled trial in low-income patients in Argentina showed that a community health worker-led multicomponent intervention emerge significant effects both in reducing blood pressure level and improving medication adherence [26].

In 2016, China's State Council Medical Reform Office and six other ministries jointly promulgated Guidelines on Advancing Family Physician Contract Services—a strategic initiative to strengthen nationwide primary healthcare infrastructure [27]. This policy empowers community physicians to implement personalized hypertension management through precision health interventions. However, multilevel challenges persist, with system-level barriers, community-specific limitations, and patient-related factors collectively undermining blood pressure control efforts, particularly in optimizing medication adherence and lifestyle modification. These realities underscore the urgent need for an integrated care model combining structured health education with system-supported behavioral interventions. This study aims to evaluate the efficacy of multidimensional health management strategies in enhancing both medication adherence and lifestyle optimization among Chinese hypertensive populations.

## 2. Methods

### 2.1. Research Design

This analysis utilized data from the China Rural Hypertension Control Project (CRHCP), whose cluster-randomized trial design has been detailed elsewhere [24]. CRHCP was an open-label, cluster-randomized controlled trial evaluating a multicomponent intervention led by village doctors versus standard care for blood pressure management in rural China. The trial's primary objective focused on developing an evidence-based implementation framework for intensive blood pressure control in resource-limited settings [24]. Between May and November 2018, 33,995 participants from 326 villages (163 intervention/163 control) across three geographically diverse provinces (Liaoning, Shaanxi, and Hubei) were enrolled. This secondary analysis focused exclusively on pilot-phase data due to operational constraints in data availability during the early implementation stage.

### 2.2. Participants

Participants were required to meet the following criteria: (1) age ≥ 40 years; (2) untreated systolic BP ≥ 140 mmHg or untreated diastolic BP ≥ 90 mmHg (with specific thresholds of ≥ 130/80 mmHg for individuals with established cardiovascular disease, diabetes, or chronic kidney disease); (3) active enrollment in China's New Rural Cooperative Medical Scheme (NRCMS)—the national insurance program covering 99% rural populations for essential healthcare [28]; (4) nonpregnant status with no pregnancy plans; and (5) capacity to provide written informed consent. Full exclusion criteria (e.g., terminal illness and secondary hypertension) are detailed in the primary trial publication [24]. Written informed consent was obtained from all participants.

### 2.3. Procedure

Guided by the WHO's Innovative Care for Chronic Conditions (ICCC) framework, this study formulated a multilevel implementation strategy tailored to China's healthcare ecosystem. Distinguished from standard care, this intervention systematically integrates three interdependent components: (1) health-system strengthening through a three-tiered governance structure (municipal/county ⟶ township ⟶ village); (2) provider capacity building via protocolized training; and (3) patient-centered empowerment with digital health tools. The model operationalizes chronic disease management across administrative levels through standardized task-shifting protocols and interprofessional collaboration networks.

#### 2.3.1. Healthcare Systems

According to the performance appraisal standards for hypertension patient management under the National Basic Public Health Service Project, village doctors are evaluated based on routine management (e.g., blood pressure monitoring and lifestyle guidance) combined with key indicators such as patient medication adherence, hypertension control rate, standardized follow-up rate, and blood pressure adherence rate. These metrics are integrated into a comprehensive performance evaluation system for village doctors, supported by a well-defined performance assessment and incentive mechanism. To streamline this process, the health system employs a hypertension management app (administrative portal) with embedded modules that track village doctors' participation in health education and hypertension management, including the frequency of interventions and patient feedback scores. This enables real-time online performance monitoring. In addition, through the administrative portal of the app, the competent health authority can establish and manage virtual physician teams, facilitating jurisdiction-specific supervision and evaluation across different villages. The platform also allows dynamic allocation and optimization of medical resources based on real-time feedback from subordinate healthcare teams.

#### 2.3.2. Village Doctors

Village doctors are responsible for the routine management of hypertensive patients in accordance with the 2018 Chinese Guidelines for Prevention and Treatment of Hypertension and the National Clinical Practice Guidelines on the Management of Hypertension in Primary Health Care in China (2020) [3, 12]. Their duties include standardized blood pressure measurement, antihypertensive drug prescription and adjustment, lifestyle modification counseling, medication adherence guidance, and patient follow-up. Prior to the study, village doctors received standardized training covering proper blood pressure measurement techniques, a tiered hypertension management protocol, and lifestyle intervention strategies (e.g., smoking cessation, alcohol reduction, dietary sodium restriction, and increased physical activity). Using a hypertension management app (physician portal), village doctors oversee hypertensive patients within their jurisdiction while being subject to supervision and evaluation by higher-level health authorities. Daily online check-ins via the app enable village doctors to monitor patients' blood pressure trends and medication adherence in real time, allowing for timely remote guidance. For patients with poor adherence, village doctors employ a comprehensive management approach through the app, supplemented by telephone/WeChat communication, addressing blood pressure control, medication compliance, health literacy, behavioral attitudes, and lifestyle modifications. Furthermore, village doctors maintain close collaboration with superior medical departments. In cases of complex clinical scenarios or therapeutic uncertainties, they consult with senior physicians to ensure treatment appropriateness and patient safety throughout the care process.

#### 2.3.3. Patients

Study participants received standardized hypertension health education, including instruction on the importance of blood pressure control, proper techniques for self-measured blood pressure monitoring, and evidence-based lifestyle modifications. Patients were instructed to measure their blood pressure at least weekly and record measurements through the patient portal of the hypertension management mobile application, enabling real-time monitoring by village healthcare providers. To enhance treatment adherence, we established patient support networks involving family members. These social support groups played an active role in hypertension management by (1) reminding patients to perform regular blood pressure monitoring, (2) ensuring timely medication administration, and (3) reinforcing long-term self-management behaviors. Family members received specific training to effectively support patients in maintaining these health behaviors, creating a structured home environment conducive to blood pressure control.

### 2.4. Factors and Outcomes

Baseline and follow-up data were collected through standardized questionnaires. Demographic and clinical variables included age, sex, ethnicity, body mass index (calculated from measured weight and height), smoking status, alcohol consumption, history of diabetes mellitus, chronic kidney disease, and current antihypertensive medication classes (diuretics, β-blockers, calcium channel blockers, ACE inhibitors, ARBs, or other agents). Following the American Heart Association protocol [29], certified village doctors obtained three consecutive blood pressure measurements at each assessment using standardized techniques. Medication adherence was measured by the following questions: (1) How many days have you not taken any antihypertensive drugs in the past 7 days? (2) How many days have you taken antihypertensive drugs less than the prescribed dose in the past 7 days? (3) Have you ever forgotten or taken less antihypertensive drugs? and (4) Sometimes if you feel worse when taking medicine, will you stop taking medicine? The answer to all the above questions is “no,” which is regarded as good medication adherence. In accordance with guideline recommendations [3, 12] and established methodology [15, 26, 30], the adherence of healthy lifestyle was evaluated by using a self-designed questionnaire, including whether to reduce smoking, reduce alcohol consumption, reduce salt intake, maintain normal weight, increase the intake of vegetables and fruits, and increase physical activities, respectively.

### 2.5. Statistical Analysis

Descriptive statistics characterized participants' baseline characteristics, compliance patterns, and lifestyle behavior modifications. Continuous variables were presented as the mean ± standard deviation (SD), while categorical variables were expressed as frequencies (percentages). To assess between-group differences in the proportion of patients achieving good adherence, we employed a generalized linear mixed-effects model with two-tailed testing (*α* = 0.05). Treatment effects were estimated with 95% confidence intervals (CIs). Secondary analyses adjusted for clinically relevant covariates including age, sex, educational attainment, and baseline body mass index. Prespecified subgroup analyses stratified by age, gender, education level, and comorbidity status were additionally performed. Missing data were handled using multiple imputation via the Markov chain Monte Carlo (MCMC) method. Initial data processing utilized Microsoft Excel 2016 (Microsoft Corp., Redmond, WA), with primary statistical analyses conducted in SAS 9.4 (SAS Institute Inc., Cary, NC). Forest plots were generated using the “forestplot” package in R 4.2.1 (R Foundation for Statistical Computing). All statistical tests were two-sided, with *p* values < 0.05 considered statistically significant.

## 3. Results

From May 8 to November 28, 2018, a total of 9204 participants were recruited into this study, with 4223 from the intervention group and 4981 from the control group. The last 18-month follow-up visit was conducted in August, 2020. During the 18 months, 540 patients in the intervention group and 963 patients in the control group were lost to follow-up. Baseline characteristics of study participants between two groups are shown in Table 1. Among the participants, 47.7% (*n* = 4393) aged 65 or above, 60.2% (*n* = 5537) were female, 92.8% (*n* = 8545) were Han, 29.3% (*n* = 2699) were not educated, and 83.2% (*n* = 7655) were married. Only less than one third (29.6%) of the participants had normal BMI. Of the 9204 participants, 22.8% (*n* = 2098) were current smoker, 17.6% (*n* = 1616) were current drinker, 21.3% (*n* = 1956) had a history of major cardiovascular disease, 15.4% (*n* = 1416) had a history of hyperlipidemia, 10.4% (*n* = 958) had a history of diabetes, and 0.7% (*n* = 62) had a history of chronic kidney disease.

Baseline adherence statuses of study participants were similar between the two groups.

At the baseline, the proportion of individuals with good medication adherence was 45.5% and the proportion of individuals who adhered to more than 3 healthy lifestyles was 29.7%. The average systolic blood pressure of the subjects was 155.99 mmHg (SD = 19.27), 156.45 mmHg (SD = 19.58) in the intervention group and 155.6 mmHg (SD = 19.00) in the control group. The average diastolic blood pressure of the subjects was 86.56 mmHg (SD = 11.62), 86.81 mmHg (SD = 11.74) in the intervention group and 86.35 mmHg (SD = 11.51) in the control group. There was no significant difference in the control rate of hypertension between the two groups. The specific results are shown in Table 2.

At 18 months, 2265 (61.5%) of 3683 patients in the intervention group and 2270 (56.5%) of 4018 patients in the control group had good medication adherence, with a group difference of 5.0% (95% CI: 2.8–7.2; *p* < 0.001). Likewise, 1687 (45.8%) of 3683 patients in the intervention group and 1354 (33.7%) of 4018 patients in the control group could adhere to more than 3 healthy lifestyles, with a group difference of 12.1% (95% CI: 9.9–14.3; *p* < 0.001). In terms of average blood pressure levels, mean systolic blood pressure was 143.66 mmHg in the intervention group and 153.4 mmHg in the control group, with a group difference of −9.74 mmHg (95% CI: −10.57–−8.91; *p* < 0.001); mean diastolic blood pressure was 79.77 mmHg in the intervention group and 83.76 mmHg in the control group, with a group difference of −3.99 mmHg (95% CI: −4.50–−3.48; *p* < 0.001). At 18 months, 1591 (43.2%) of 3683 patients in the intervention group and 960 (23.9%) of 4018 patients in the control group had a systolic blood pressure of less than 140 mm Hg and a diastolic blood pressure of less than 90 mm Hg, with a group difference of 19.2% (95% CI: 17.2–21.3; *p* < 0.001). The specific results are shown in Table 3.

**Figure 1:**
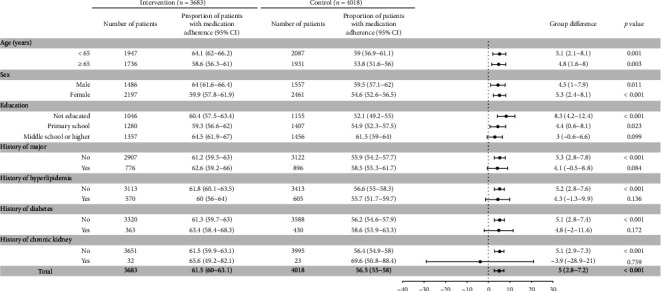
Difference in the proportion of patients with medication adherence at 18 months by subgroups.

**Figure 2:**
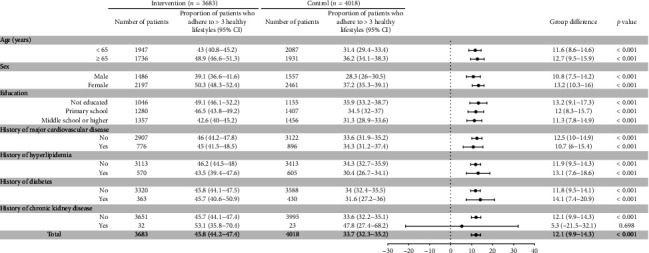
Difference in the proportion of patients with more than 3 healthy lifestyles at 18 months by subgroups.

Differences in the proportion of the outcomes in selected prespecified subgroups are provided in Figures 1 and 2; the findings show a consistent benefit with comprehensive health management measures. The differences of medication adherence at 18 months between the intervention and control groups were consistent by age and gender. Compared with the control group, patients with lower education level and no comorbid history had better medication adherence. The differences of adhere to healthy lifestyles, including age, gender, education, and comorbid history, were also consistent across subgroups.

## 4. Discussion

This cluster-randomized controlled trial demonstrated that comprehensive health management strategies effectively improved both medication adherence and healthy lifestyle behaviors among hypertensive patients in rural Northeast China. Specifically, the intervention group showed significant reductions in smoking, alcohol consumption, and salt intake, while demonstrating increased consumption of vegetables and fruits and enhanced engagement in regular physical activity compared with the control group.

These findings hold significant public health implications for hypertension management. While extant studies have established that enhanced medication adherence and lifestyle modifications can substantially mitigate hypertension risks [31–33], and while cost-effective antihypertensive medications and lifestyle education programs are universally accessible in China, the nation continues to grapple with alarmingly low adherence rates [26]. A critical barrier persists in the absence of sustainable implementation frameworks to address systemic challenges in hypertension control [26]. For community-dwelling hypertensive populations, prioritizing adherence optimization and lifestyle transformation becomes paramount when basic healthcare services are accessible [34]. Consequently, scaling up this evidence-based intervention in regions with established healthcare infrastructure could substantially improve therapeutic adherence among hypertensive residents, thereby reducing uncontrolled hypertension and its cardiovascular complications.

Prior intervention studies have demonstrated the efficacy of various strategies—including simplified medication regimens, eHealth technologies, structured patient education, performance-based incentives, and behavioral counseling—in enhancing medication adherence and promoting lifestyle modifications [35–39]. Healthcare provider engagement has been identified as a critical determinant of antihypertensive treatment compliance, with clinician training programs proving particularly effective in optimizing therapeutic outcomes [40]. A narrative review of 42 studies revealed that patient-focused behavioral interventions showed stronger associations with improved medication adherence compared to provider-targeted or system-level interventions [41]. Village doctors offer unique advantages in delivering primary healthcare services to rural populations. As community members residing alongside their patients, they develop stronger trust-based relationships with the hypertensive individuals under their care. This unique position not only enables them to provide more accessible medical services during management but also allows for tailored health guidance on lifestyle modifications and medication adherence based on individual patient and family circumstances.

Previous research has identified multiple factors influencing medication adherence and healthy lifestyle behaviors in hypertensive patients, encompassing socioeconomic, healthcare system-related, treatment-related, disease-related, and patient-related dimensions [33, 42–44]. Interventions targeting these factors have consistently shown positive impacts on hypertension management adherence. Notably, the comprehensive health management model developed in our study represents an innovative departure from single-intervention approaches. By leveraging digital health technologies, it dismantles traditional barriers between patients, healthcare teams, and health systems, creating an integrated tripartite communication framework. This approach offers a potentially superior paradigm for implementing patient-centered, comprehensive hypertension management.

Recent evidence has shown the feasibility, acceptability, and success of digital interventions in increasing medication adherence and modifying the lifestyle behaviors of patients with hypertension [36, 45–47]. In these studies, digital interventions take mobile apps or portable intelligent devices as their carriers to provide services such as blood pressure tracking, medication reminders, patient education, and abnormal value warnings. In our research, by developing different portals in the hypertension management app, we embedded additional functions for different service providers alongside implementing the above functions, such as the patient portal's feedback scoring module. With the increasing popularity of mobile electronic devices, this method is expected to provide new approaches for the health management of hypertensive patients, especially for improving adherence.

The subgroup analysis in this study revealed significant educational disparities in medication adherence improvement (*p* < 0.05), aligning with previous research findings [30, 48]. Specifically, patients with lower educational attainment demonstrated poorer medication adherence compared with their more educated counterparts, primarily attributable to limited health literacy. Notably, our intervention produced more pronounced adherence improvements in the less-educated subgroup, suggesting that health education effectively compensates for baseline knowledge deficits. These findings underscore the importance of implementing tailored, literacy-appropriate intervention strategies in population-based hypertension management programs, with particular emphasis on vulnerable subgroups.

Several limitations should be noted when interpreting our study findings. First, the lifestyle behavior changes in this study were assessed through self-reported data, which may introduce reporting bias. Second, there were methodological differences in data collection between the pandemic longitudinal study and the present study. All baseline data were obtained via telephone interviews during the peak of the COVID-19 pandemic, a circumstance that inevitably influenced the results. Recent literature indicates that COVID-19 significantly impacted lifestyle modifications among hypertensive patients [49]. For instance, physical activity analysis may reflect artificially reduced levels due to China's stringent lockdown policies at the time, potentially leading to overestimated intervention effects. Therefore, future studies should complement self-reports with objective behavioral measures, particularly for physical activity assessment [50]. In addition, nationwide health promotion campaigns like the Healthy China Initiative may have contributed to improved medication adherence and lifestyle changes in the general population. Although our study demonstrates that comprehensive health management significantly enhances medication adherence and lifestyle behaviors among hypertensive patients, sustained behavioral change ultimately depends on patients' intrinsic motivation rather than researcher-imposed interventions. Further well-designed controlled trials are needed to evaluate the effects of integrated health management strategies on compliance behaviors and blood pressure control, which would strengthen the evidence base for large-scale program implementation.

## 5. Conclusions

This study demonstrates that comprehensive health management interventions significantly improved medication adherence and promoted healthier lifestyle behaviors among rural Chinese patients with hypertension. These findings underscore the potential of structured health management programs in resource-limited settings. Future research should evaluate the cost-effectiveness and generalizability of such interventions to optimize their implementation in diverse populations.

## Figures and Tables

**Table 1:** Baseline characteristics of study participants.

Characteristics of participants	All participants (*n* = 9204)	Intervention (*n* = 4223)	Control (*n* = 4981)	*p* value
Age (years)				0.248
< 65	4811 (52.3)	2235 (52.9)	2576 (51.7)	
≥ 65	4393 (47.7)	1988 (47.1)	2405 (48.3)	
Sex, *n* (%)				0.315
Male	3667 (39.8)	1706 (40.4)	1961 (39.4)	
Female	5537 (60.2)	2517 (59.6)	3020 (60.6)	
Ethnicity, *n* (%)				0.001
Han	8545 (92.8)	3893 (92.2)	4652 (93.4)	
Manchu	473 (5.1)	252 (6)	221 (4.4)	
Mongolian	158 (1.7)	72 (1.7)	86 (1.7)	
Others	28 (0.3)	6 (0.1)	22 (0.4)	
Residential status, *n* (%)				< 0.001
Living alone or with spouse only	5431 (59)	2423 (57.4)	3008 (60.4)	
Live with children	3398 (36.9)	1680 (39.8)	1718 (34.5)	
Others	375 (4.1)	120 (2.8)	255 (5.1)	
Education, *n* (%)				0.528
Not educated	2699 (29.3)	1214 (28.7)	1485 (29.8)	
Primary school	3161 (34.3)	1459 (34.5)	1702 (34.2)	
Middle school or higher	3344 (36.3)	1550 (36.7)	1794 (36)	
Marital status, *n* (%)				0.398
Married/cohabiting	7655 (83.2)	3530 (83.6)	4125 (82.8)	
Widower/widow	1366 (14.8)	617 (14.6)	749 (15)	
Divorce/live apart/single	183 (2)	76 (1.8)	107 (2.1)	
BMI, *n* (%)				0.681
BMI < 18.5	144 (1.6)	68 (1.6)	76 (1.5)	
18.5 ≤ BMI ≤ 23.9	2720 (29.6)	1268 (30)	1452 (29.2)	
23.9 < BMI ≤ 27.9	3893 (42.3)	1759 (41.7)	1319 (26.5)	
BMI > 27.9	2447 (26.6)	1128 (26.7)	4981 (100)	
Cigarette smoking, *n* (%)				0.891
Never smoked	6173 (67.1)	2843 (67.3)	3330 (66.9)	
Former smokers	933 (10.1)	424 (10)	509 (10.2)	
Current smokers	2098 (22.8)	956 (22.6)	1142 (22.9)	
Alcohol drinking, *n* (%)				0.251
Never drink	6931 (75.3)	3148 (74.5)	3783 (75.9)	
Former drinker	657 (7.1)	317 (7.5)	340 (6.8)	
Current drinker	1616 (17.6)	758 (17.9)	858 (17.2)	
Personal salt intake(g/month), (mean ± SD)	236.16 ± 132.12	236.25 ± 133.75	236.08 ± 130.67	0.956
History of major cardiovascular disease^∗^, *n* (%)				0.626
No	7248 (78.7)	3316 (78.5)	3932 (78.9)	
Yes	1956 (21.3)	907 (21.5)	1049 (21.1)	
History of hyperlipidemia, *n* (%)				0.127
No	7788 (84.6)	3547 (84.0)	4241 (85.1)	
Yes	1416 (15.4)	676 (16.0)	740 (14.9)	
History of diabetes, *n* (%)				0.558
No	8246 (89.6)	3792 (89.8)	4454 (89.4)	
Yes	958 (10.4)	431 (10.2)	527 (10.6)	
History of chronic kidney disease, *n* (%)				0.156
No	9142 (99.3)	4189 (99.2)	4953 (99.4)	
Yes	62 (0.7)	34 (0.8)	28 (0.6)	
Numbers of antihypertensive drugs taken daily, *n* (%)				< 0.001
0	2939 (31.9)	1184 (28)	1755 (35.2)	
1	5209 (56.6)	2504 (59.3)	2705 (54.3)	
2	938 (10.2)	481 (11.4)	457 (9.2)	
≥ 3	118 (1.3)	54 (1.3)	64 (1.3)	
Monthly expenditure on hypertension(CNY), (mean ± SD)	37.29 ± 51.51	38.96 ± 56.4	35.72 ± 46.39	0.017

^∗^Major cardiovascular disease includes hyperlipidemia, myocardial infarction, stroke, and heart failure.

**Table 2:** Baseline adherence status of study participants.

	All participants (*n* = 9204)	Intervention (*n* = 4223)	Control (*n* = 4981)	*p* value
Medication adherence (95% CI)	45.5 (44.5∼46.6)	45.8 (44.3∼47.3)	45.3 (43.9∼46.7)	0.612
Healthy lifestyle behaviors (95% CI)				
Nonsmoker or former smoker	77.2 (76.3∼78.1)	77.4 (76.1∼78.6)	77.1 (75.9∼78.2)	0.742
No or limited alcohol intake	82.4 (81.7∼83.2)	82.1 (80.9∼83.2)	82.8 (81.7∼83.8)	0.363
Normal weight	29.6 (28.6∼30.5)	30 (28.6∼31.4)	29.2 (27.9∼30.4)	0.359
Reduce salt intake	29 (28.1∼29.9)	29.1 (27.8∼30.5)	28.9 (27.7∼30.2)	0.82
Engaged in recommended amount of physical activity	60.9 (59.9∼61.9)	61.8 (60.4∼63.3)	60 (58.7∼61.4)	0.078
Consumed recommended amount of fruits or vegetables	40.6 (39.6∼41.6)	41.2 (39.7∼42.7)	40.1 (38.7∼41.4)	0.261
≤ 3 behaviors	70.3 (69.3∼71.2)	70 (68.6∼71.4)	70.5 (69.2∼71.8)	0.577
> 3 behaviors	29.7 (28.8∼30.7)	30 (28.6∼31.4)	29.5 (28.2∼30.8)	0.577
Average SBP^∗^ (mmHg), (mean ± SD)	155.99 ± 19.27	156.45 ± 19.58	155.6 ± 19	0.034
Average DBP^a^ (mmHg), (mean ± SD)	86.56 ± 11.62	86.81 ± 11.74	86.35 ± 11.51	0.06
Hypertension control rate (95% CI)	17.7 (16.9∼18.5)	17 (15.9∼18.2)	18.2 (17.2∼19.3)	0.133

^∗^SBP, systolic blood pressure.

^a^DBP, diastolic blood pressure.

**Table 3:** Intervention effect on adherence and blood pressure outcomes.

	All participants (*n* = 7701)	Intervention (*n* = 3683)	Control (*n* = 4018)	Group difference	*p* value
Medication adherence (95% CI)	58.9 (57.8∼60)	61.5 (60∼63.1)	56.5 (55∼58)	5 (2.8∼7.2)	< 0.001
Healthy lifestyle behaviors (95% CI)					
Nonsmoker or former smoker	79.6 (78.7∼80.5)	85.6 (84.4∼86.7)	74 (72.7∼75.4)	11.5 (9.8∼13.3)	< 0.001
No or limited alcohol intake	87.9 (87.2∼88.6)	93.8 (93∼94.5)	82.5 (81.3∼83.7)	11.3 (9.8∼12.7)	< 0.001
Normal weight	30.4 (29.4∼31.4)	35.8 (34.3∼37.4)	25.4 (24.1∼26.8)	10.4 (8.4∼12.5)	< 0.001
Reduce salt intake	22.4 (21.5∼23.4)	23.4 (22∼24.8)	21.6 (20.3∼22.8)	1.9 (0∼3.7)	0.052
Engaged in recommended amount of physical activity	47.6 (46.5∼48.7)	48.7 (47.1∼50.4)	46.6 (45.1∼48.2)	2.1 (-0.1∼4.4)	0.063
Consumed recommended amount of fruits or vegetables	45.6 (44.5∼46.7)	47 (45.4∼48.6)	44.3 (42.7∼45.8)	2.8 (0.5∼5)	0.015
≤ 3 behaviors	60.5 (59.4∼61.6)	54.2 (52.6∼55.8)	66.3 (64.8∼67.7)	−12.1 (-14.3∼-9.9)	< 0.001
> 3 behaviors	39.5 (38.4∼40.6)	45.8 (44.2∼47.4)	33.7 (32.3∼35.2)	12.1 (9.9∼14.3)	< 0.001
Average SBP^∗^ (mean ± SD)	148.74 ± 19.32	143.66 ± 17.82	153.4 ± 19.46	−9.74 (-10.57∼-8.91)	< 0.001
Average DBP^a^ (mean ± SD)	81.85 ± 11.60	79.77 ± 10.94	83.76 ± 11.86	−3.99 (-4.50∼-3.48)	< 0.001
Hypertension control rate (95% CI)	33.1 (32.1∼34.2)	43.2 (41.6∼44.8)	23.9 (22.6∼25.3)	19.2 (17.2∼21.3)	< 0.001

^∗^SBP, systolic blood pressure.

^a^DBP, diastolic blood pressure.

## Data Availability

The data that support the findings of this study are available from the Ministry of Science and Technology of China. Restrictions apply to the availability of these data, which were used under license for this study. Data are available from the authors with the permission of Ministry of Science and Technology of China.
